# Analyzing Gender Differences in the Relationship between Family Influences and Adolescent Offending among Boys and Girls

**DOI:** 10.1007/s12187-016-9435-6

**Published:** 2016-12-16

**Authors:** E-L. Nilsson

**Affiliations:** Faculty of Health and Society, Malmö University, 205 06 Malmö, Sweden

**Keywords:** Adolescent offending, Gender differences, Family influences, Social bonding

## Abstract

The aim of this paper is to examine gender differences in several dimensions of family-related variables in the explanation of adolescent offending. Analyses are conducted to examine: 1) whether boys and girls differ in levels of offending and in levels of family variables respectively, 2) whether the correlations between family variables and offending differ by gender, and 3) whether the family variables explain gender differences in adolescent offending. The study employs self-report data from a sample of young adolescents in Halmstad, Sweden (*N* = 889). The findings show that boys offend more than girls and that there are clear differences in both levels of the family variables and in the associations between the different family variables and offending. The findings also show that the family variables cannot explain gender differences in offending. The findings in this study are not only relevant theoretically, they also have important policy implications.

## Introduction

There is strong evidence that the family environment has a major influence on the present and future development of children’s behavior and well-being. These findings have been presented across different cultures, contexts and countries (see e.g., Lee and Yoo 2015; Viner et al. 2012). A substantial amount of research aimed at explaining gender differences in young people’s well-being and in unhealthy behaviors, such as adolescent offending, has looked to the family situation for explanations (e.g., Levin et al. 2012; Worthen 2011).

When it comes to adolescent offending, it is well established that girls present lower levels of offending in both official statistics and self-report data (e.g., Moffitt et al. 2001; Smith 2014; Weerman et al. 2015). Even though the family has repeatedly been identified as an important factor in the understanding of offending among both boys and girls (e.g., Hoeve et al. 2009; Junger-Tas et al. 2004; Kruttschnitt and Giordano 2009), studies focused on gender differences in the relationship between the family and adolescent offending have produced inconsistent findings. It is unclear whether the same family variables can explain the offending of boys and girls respectively and also whether family influences have a different significance for boys and girls (Hoeve et al. 2009, 2012; Wong et al. 2010).

Within this field of research, most previous studies have focused on measures of attachment to parents and/or parental monitoring, and there is a lack of studies that have also included additional variables. The current study therefore includes a range of family variables representing elements of family attachment that have to date not been included in a single analysis conducted for boys and girls. In relation to much of the research dealing with the family and adolescent offending, which is characterized by a unidimensional attached-unattached conception of family relationships for example, this study will contribute additional knowledge to our understanding of family influences on offending among boys and girls. It may be particularly relevant and interesting to study this issue in a Swedish context, where the gender gap in offending has declined over recent decades (Estrada et al. 2015), and where the family environment and parenting practices are often described as being democratic and characterized by gender equality (Ahlberg et al. 2008; Trifan et al. 2014).

## Background

To begin with a presentation of the study’s theoretical framework, the field of criminology contains numerous perspectives and theories that have been advanced in the study of gender differences in the relationship between the family and adolescent offending. However, the most commonly applied theory is Social bonding theory (Hirschi 1969). As is the case in all control theories, social bonding theory does not strive to explain offending per se, but rather to explain conformity. According to control theorists, controls or restraints are what keep individuals from offending (Hirschi 1969; Sampson and Laub 1993). In social bonding theory, Hirschi posits the social bonds as the source of social control and conformity, and presents four such controlling bonds that bind a person to society: *Attachment,* which refers to the affection and emotions an individual holds for significant others, *commitment* to conventional pursuits, *involvement* in conventional activities and *belief*, which refers to an individual’s bonds to society. It is theorized that the propensity to commit crime increases when an individual’s bonds to society are weakened or broken (Hirschi 1969).

This theory, with its focus on family-related constructs of relevance to the attachment between parent and child, will constitute the theoretical framework for the current study. Of the different elements described in social bonding theory, attachment is generally described as the most important. In particular, attachment to one’s parents has been shown to be one of the most essential controlling factors (Hirschi 1969). According to this theory, weak social ties to parents will increase the likelihood of adolescent offending, and one important reason for committing offences is that those with weak social ties feel that they have nothing to lose.

Attachments between parents and children manifest themselves on a number of different dimensions. However, all of these dimensions play a significant role when it comes to understanding the strength of the parent-child bond. For example, an attached child is used to sharing his or her life with parents (Hirschi 1969). Communication, but perhaps most importantly, the child’s free disclosure of information to parents, has been identified as an indicator of a good parent-child relationship (Kerr and Stattin 2000). Hirschi (1969) also states that children who are attached to their parents are more likely to spend time in the presence of their parents. Further, troubled parent-child relationships, such as relationships characterized by many parent-child conflicts, may result in a variety of negative consequences, including the weakening of positive parent-child bonding, and adolescent offending (Choi et al. 2008). In the original statement of social bonding theory, attachments between parent and child, and their correlation with offending, were proposed to be universal with respect to gender (Hirschi 1969).

When it comes to empirical findings, it has been noted that girls overall present higher levels of family-related protective factors. For example, the social control of girls appears to be stronger and more intensive than is the case for boys, which would explain why girls’ levels of offending are lower than those of boys (e.g., Junger-Tas et al. 2004; Svensson 2003; Weerman et al. 2015). In addition, the literature mainly shows that girls have stronger bonds with their parents (e.g., Svensson 2003), and that they disclose more information to their parents about their lives (e.g., Stattin and Kerr 2000). Girls also seem to spend more time at home, which is seen as a protective factor, mainly because it limits the opportunities for offending (Hirschi 1969). A similar explanation that has also been suggested is that girls’ offending is less common as a result of them being less exposed to risk factors than boys (Fagan et al. 2007; Moffitt et al. 2001). In the literature, it has been suggested that boys spend more time with friends without adult supervision (Weerman et al. 2015; Weerman and Hoeve 2012), which has also been shown to be associated with offending (Svensson and Oberwittler 2010). Thus, less time spent with the family increases the likelihood of spending more time with friends (Warr 2005).

As regards gender differences in the correlations between family variables and adolescent offending, the results presented in previous research are somewhat more complicated and contradictory. Recurrent findings show that overall, weak family attachments are important correlates of offending for both boys than girls (e.g., Hoeve et al. 2009; Junger-Tas et al. 2004; Kruttschnitt and Giordano 2009). It has also been suggested that gender differences in a range of unhealthy adolescent behaviors, including for example the fact that boys present higher levels of aggressive behavior and violence than girls (Loeber et al. 2013; Weerman et al. 2015), arise because of differences between boys and girls in parental attachments (Mandara et al. 2012). However, even after reviewing the literature, it remains rather unclear whether boys and girls bond with their parents in the same way, or whether different dimensions of parent-child attachment have the same importance for behavioral outcomes, such as offending, for boys and girls respectively (see e.g. Hoeve et al. 2009, 2012).

Looking to research findings on the links between offending and different dimensions of family attachment among boys and girls, some studies have found that girls are more influenced by bonds to the family than boys (Alarid et al. 2000; Heimer and De Coster 1999) and that girls are more likely to bond with their parents in ways that prevent or preclude offending (Worthen 2011). By contrast, other studies have found parental attachment to have more of an influence on boys’ offending (e.g., Anderson et al. 1999). Another group of researchers have found very few gender differences in the effects of family attachment on adolescent offending (Hubbard and Pratt 2002; Loeber and Stouthamer-Loeber 1986).

In the literature, girls’ offending has more frequently been associated with openness towards parents (e.g., Wong et al. 2010), while boys’ offending has more often been found to be associated with growing up in a single parent household. In addition, boys are generally more vulnerable to the negative influence of peers (Weerman and Hoeve 2012). When it comes to parental monitoring, a number of studies have found that poor parental monitoring has a similar effect for both boys and girls (Martens 1997). Other researchers, however, have found that the effect is stronger for boys than girls (Cernkovich and Giordano 1987), while still others have primarily found an association with boys’ offending (e.g., Heimer and De Coster 1999). Conflicts between parents and children have been found to be associated with offending among both boys and girls (Wong et al. 2010).

In addition to the inconsistent findings described above, it is also important to consider some of the difficulties associated with the variables employed within this research field. The definitions and measurements of common variables have been shown to be far from straightforward; they vary across, and also within research fields. For example, as has been noted above, the most commonly used variables are different measures of attachment and parental monitoring (e.g. Chapple et al. 2005; Cota-Robles and Gamble 2006; Worthen 2011). However, the way these variables have been measured and defined, particularly monitoring, differs considerably between different studies (see Stattin and Kerr 2000). This not only makes it difficult to summarize and compare results, but it might also have implications for the results themselves. Hoeve et al. (2009) have suggested that the strength of the link between parenting and adolescent offending may be influenced by these difficulties. Chapple et al. (2005) have also argued that the inconsistent findings reported by the research may be a consequence of measurement problems.

Further, a substantial part of the research has analyzed different measures of social bonding and/or social control. Generally speaking, there is a strong tradition within this body of research of studying family relationships separately from parenting practices (Hoeve et al. 2009; Kerr and Stattin 2000). It is relatively unusual to find studies that include a variety of different variables in the same analysis. Worthen (2011), however, did include a variable measuring time spent with parents in addition to measures of both emotional attachment and parental monitoring. Moffitt et al. (2001) included a wide variety of variables in their study, and in addition to measures of attachment and monitoring, they also considered a number of variables that are more rarely employed, including for example harsh and inconsistent discipline. However, in the study in question, these different variables were measured at different ages, which might have implications for the results, since there is research showing that family variables produce larger effect sizes in samples of younger children as compared with samples of older children (Hoeve et al. 2012). Altogether, there are indications that variations in measurement may help to account for some of the inconsistent results in this area.

In summary, much has been written about gender differences in the associations between different dimensions of family attachment and adolescent offending. It is recognized that the family is important for understanding offending among both boys and girls. It is also clear that different results have been presented within the field and that the constructs and measures employed differ across studies, which means that there is no consistent and straightforward answer to the question of how gender may affect the influence of dimensions of family attachments on adolescent offending. The findings are both inconsistent and scarce, and there is a need for studies to incorporate several different family variables in order to be able to better understand the dynamics that shape children’s family environment and development (Fosco et al. 2012).

## The Present Study

Against this background, the aim of this paper is to examine gender differences in several dimensions of family-related variables in the explanation of adolescent offending. Using the theoretical framework provided by social bonding theory, with a focus on the dimension of attachment between parent and child, the following family variables are included in the study: parental monitoring, conflict with parents, attachment to parents, child disclosure and time spent with friends. To our knowledge, no previous study has included all of these variables in the same analysis. Furthermore, the objective of the study is also to expand on prior research by including several dimensions of family-related variables in the analyses in order to provide a more nuanced presentation of the correlations between family influences and adolescent offending among boys and girls. Three research questions will be examined: 1) Do boys and girls differ in levels of offending and levels of family variables respectively? 2) Do the correlates of offending differ by gender? 3) Do family variables explain gender differences in adolescent offending?

## Method

### Sample

The study employs data from the Halmstad school survey (e.g., Pauwels and Svensson 2008; Svensson and Pauwels 2010; Svensson et al. 2010). Halmstad is a medium-sized city on the south-west coast of Sweden with approximately 90,000 inhabitants. As regards levels of income, unemployment, and educational achievement, the sample is comparable to the Swedish average. The Halmstad school survey included all students in their final year of compulsory education (*M* age = 15.14 years, age range: 14–16, *SD* = 0.41). The study constitutes a census of 1003 adolescents in 13 schools. In Halmstad, the headmaster of each school distributed the questionnaires with information about the study to teachers, and students completed the questionnaires during lesson time in the presence of the teacher. In line with Swedish recommendations on research ethics, the students answering the questionnaire were guaranteed complete anonymity. Since self-report data are prone to the possibility of error through non-response, as well as the under-reporting and over-reporting of offending (e.g., Junger-Tas and Marshall 1999), each student was provided with an envelope in which to place the completed questionnaire in order to reduce the effects associated with these issues.

The non-response rate for the population was 15.2%. Following listwise deletion of missing values, the analyses presented below are based on 889 respondents. The Halmstad survey sample was comprised of 48.4% boys and 51.6% girls. Of the respondents, 74.2% had a completely native background (both parents from Sweden), 25.8% of the respondents had at least one parent with an immigrant background. A substantial majority of the respondents, 73.7% lived with two parents and 26.3% lived in a single parent family.

### Measurement of Variables

#### Dependent Variable

*Self-reported offending* is measured by a scale that sums the respondents’ offending across 14 offences. Students were asked to respond to each item independently and answered the following questions about how many times during the past year they had committed any of the following offences: shoplifting / stealing from school / bicycle theft / moped theft / car theft / theft from a car / burglary / robbery / hit someone hard / hurt someone with a knife / taken a knife with them as a weapon when going out / vandalism / tags / graffiti. The response alternatives for all of these items range from never – more than 10 times. The specific response scale options were: *never / once / 2–3 times / 4–5 times / 6–10 times / more than 10 times.* The scale has a Cronbach’s alpha of .84 (boys: .86, girls: .74). High scores on the scale imply a high frequency of offending. Since there are potential problems as a result of the skewed nature of the crime variable, a transformed crime variable log (+1 value of offence frequency scale) has been used in the analyses. The lowest observation on this scale is 0, and the highest observation is 4 (*M* = .87; *SD* = .99 ). In this sample, 46.9% of the respondents reported not having committed any criminal offences in the course of the past year. A variety scale was also created in order to test the validity of the results. The same results were obtained using the variety scale as with the frequency-based offending scale, indicating that the results presented in the study may be considered robust. In this study, the transformed frequency scale is used for all analyses.

#### Independent Variables

*Parental monitoring* is an additive scale based on five items. The respondents were asked whether they: have a curfew when to be home in the evening / need to ask for permission when going out in the evening / have to call their parents if they are going to be late home / are required to say why if they come home later than they said they would / are asked about whom they are going to be hanging out with and what they will be doing when going out in the evening. The response alternatives for these items were: *no, never / quite seldom / sometimes / quite often / yes, always*. This scale has a Cronbach’s alpha of .80 (boys: .77, girls: .79). A high score on the measure implies a high level of parental monitoring. In this study, parental monitoring measures active monitoring by parents and not parental knowledge (see Stattin and Kerr 2000).

*Conflict with parents* is an additive scale based on the following two items: how often do you have disagreements or arguments with your parents? / how often do you purposely not talk to your parents because you are mad at them? The response alternatives for these items were: *never / very seldom / 1–2 times per week / 3–5 times per week / 6–7 times per week*. The two items are strongly correlated (*r* = .65, *p* < .001), and the Cronbach’s alpha for the scale is .79 (boys: .71, girls: .81). A high score on the measure implies a high level of conflict between child and parent. These two items have been used in previous studies (e.g., Cernkovich and Giordano 1987).

*Attachment to parents* is an additive scale based on the mean values of the following five items which were asked in relation to the respondents’ relationships with both mother and father respectively: do you feel your mother/father trusts you? / do you usually feel that your mother/father gives you support and encouragement? / do you think you have a good contact with your mother/father? / do you feel that your mother/father cares about you? / are you usually able to talk to your mother/father about everything (e.g. problems)? The response alternatives for these items were: *no, never / quite seldom / sometimes / quite often / yes, always*. When the respondent answered the items for both mother and father, the construct was considered as a joint entity. There were no separate questions for students not living with their mother and/or father. The Cronbach’s alpha for this measure is .88 (boys: .86, girls: .90). A high score on the measure implies a high degree of attachment to parents. Similar items have been used in the literature (e.g., Kerr and Stattin 2000).

*Child disclosure* is an additive scale based on the following four items: Do you usually tell your parents: – which friends you hang out with? / – what you are up to when you are not at home? / – if you did something you regret? / – about how you are doing in school? The response alternatives for these items were: *no, never / quite seldom / sometimes / quite often / yes, always*. The measure has a Cronbach’s alpha of .76 (boys: .75, girls: .77). A high score on the measure implies a high level of child disclosure. These items on child disclosure have previously been used by Martens (1997) for example. This measure refers to children’s free disclosure of information about their activities, and has been identified as a primary source of parents’ knowledge (see Kerr and Stattin 2000; Stattin and Kerr 2000).

*Time spent with friends* is measured using a single item. The respondents were asked how many evenings per week they usually hang out with their friends? The response alternatives were: 0/1/2/3/4/5/6/7 *evenings per week*. A high score on the measure implies that the respondent spends a lot of time with friends. Mean score for boys: 3.72 evenings per week (SD: 1.76), mean score for girls: 3.41 evenings per week (SD: 1.86).

The *background variables* included in the study are gender, immigrant background and family structure. These background variables are assumed to be weakly correlated with offending when controls are included for family factors and most of their effect is assumed to be exerted indirectly via the family factors (e.g., Sampson and Laub 1993). *Gender* is coded as zero for boys and one for girls. *Immigrant background* is coded as zero when both parents are native Swedes, and as one if at least one of the parents was born abroad. *Family structure* is coded as zero if the respondent was living with two parents, and as one if the respondent was living in a single parent family.

Table 1 presents the correlations between the variables included in the study. The table suggests that several of the family variables are significantly related to offending in the expected direction for both boys and girls. However, immigrant background was not correlated with any of the other variables, with the exception of family structure among the girls, and will therefore not be included in the forthcoming regression analysis. There are no signs of multicollinearity, since the highest Variance of Inflation Factor score was 1.63, which is well below the acceptable threshold.

**Table 1:** Correlation matrix by gender, girls (*n* = 459) above and boys (*n* = 430) below

Variables	1.	2.	3.	4.	5.	6.	7.	8.
1. Immigrant background		.13**	.04	-.08	-.09	-.00	-.05	.10
2. Family structure	-.00		-.12	.11	-.22***	-.04	.20***	.11
3. Parental monitoring	.06	-.05		-.06	.14**	.26***	-.20***	-.18***
4. Conflict with parents	-.03	.05	-.00		-.53***	-.41***	.21***	.24***
5. Attachment to parents	-.04	-.20***	.15**	-.35***		.55***	-.19***	-.33***
6. Child disclosure	.01	-.01	.36***	-.27***	.40***		-.19***	-.29***
7. Time spent with friends	-.04	-.05	-.21***	-.03	.06	-.16***		.39***
8. Offending (log + 1)	.04	.19***	-.15***	.23***	-.19***	-.29***	.28***	

* *p* < .05. ** *p* < .01. *** *p* < .001

### Analytic Strategy

The analysis was conducted in three stages. First, differences between boys and girls in levels of offending and on the family variables were tested by means of a *t*-test for independent samples. Cohen’s *d* was used to calculate effect sizes (Cohen 1988). Second, two Ordinary Least Squares (OLS) regression analyses were conducted for boys and girls respectively. The aim of these analyses was to examine whether the relationships between the family variables and offending differ between boys and girls. A significance test was conducted to determine whether there were significant differences in the size of the coefficients between boys and girls in relation to the family variables (see Paternoster et al. 1998). Third, four OLS regression analyses were estimated in order to examine whether the family variables explain gender differences in adolescent offending. The Bonferroni correction is applied in order to avoid Type I errors due to multiple comparisons (Mundfrom et al. 2006).

## Results

### Do Boys and Girls Differ in Offending and in Levels of Family Variables?

The first stage of the presentation compares offending among boys and girls. The results indicate that boys are more involved in criminal activities than girls (see Table 2). Among boys, 60.2% have been involved in criminal activity during the past year, compared to 46.4% of the girls (χ^2^ = 17.04, *p* < .001, *t* = 6.85, *p* < .001, Cohen’s *d* = .47). The results also show significant gender differences for most of the offences examined. When the individual offence types are examined separately (table not presented), shoplifting emerges as the most commonly reported offence for both boys and girls, although boys reported a significantly higher level of involvement in shoplifting than girls (*t* = 2.21, *p* < .001). Boys were also significantly more involved in both vandalism (*t* = 5.27, *p* < .001) and theft (*t* = 4.84, *p* < .001), although the greatest gender difference is found in relation to assault offences (*t* = 6.38, *p* < .001).

**Table 2:** Gender differences in level of family dimension and offending

Variable	Boys	Girls	t Test^a^	es^b^
Parental monitoring (5–25)				
M	16.71	19.30	-8.93***	0.60
SD	4.43	4.18		
Conflict with parents (2–10)				
M	4.43	5.30	-8.00***	0.54
SD	1.44	1.76		
Attachment to parents (5–25)				
M	22.00	20.73	5.64***	0.38
SD	2.87	3.81		
Child disclosure (4–20)				
M	13.91	14.63	-3.28***	0.22
SD	3.30	3.21		
Time spent with friends (0–7)				
M	3.72	3.41	-2.50	0.17
SD	1.76	1.86		
Offending (log +1) (0–4)				
M	1.10	0.65	6.85***	0.47
SD	1.09	0.83		

^a^Based on the Bonferroni method, threshold of significance, *p* < .008.

^b^Effect size computed with Cohen’s *d* (Cohen 1988). Small effects = .2; medium effects = .5; large effects = .8. NOTE: Min-max in parentheses. High score indicates high level of exposure to family dimension/offending

Further, the results show that boys and girls differed significantly in their scores on most of the family variables. Boys reported significantly stronger attachments to parents than girls (*t* = 5.64, *p* < .001, Cohen’s *d* = .38). Girls, on the other hand, reported significantly higher levels of parental monitoring (*t* = −8.93, *p* < .001, Cohen’s *d* = .60), conflict with parents (*t* = −8.00, *p* < .001, Cohen’s *d* = .54), and child disclosure (*t* = −3.28, *p* < .001, Cohen’s *d* = .22). The scores on the time that boys and girls reported spending with friends did not differ significantly following the adjustment of the *p*-value in accordance with the Bonferroni method (*t* = −2.50, *p* = .013, Cohen’s *d* = .17).

### Do the Correlates of Adolescent Offending Differ by Gender?

Two OLS regression models were estimated to illuminate this question. Separate analyses were conducted for boys and girls in an effort to develop a more complete picture of possible gender-specific effects of the independent variables on self-reported offending. These findings are presented in Table 3. The analyses show some interesting results and important differences emerged, indicating several differences in the correlates of offending for boys and girls respectively. The results from Model 1 show that the family variable that is significantly associated with offending among the girls is attachment to parents. For boys, family structure, conflict with parents, and child disclosure are significantly associated with offending. The amount of variance explained by the model approaches 15% for both boys and girls.

**Table 3:** OLS multiple regression analysis predicting self-reported offending, by gender (unstandardised and standardised regression coefficient)

Predictors	Boys (*N* = 430)		Girls (*N* = 459)		z-value
	*b* ^a^	Beta	*b* ^a^	Beta	
Model 1					
Family structure	.44***	.17	.06	.03	2.64**
Parental monitoring	-.02	-.07	-.02	-.11	.00
Conflict with parents	.13***	.17	.04	.08	1.71
Attachment to parents	.01	.02	-.05***	-.21	2.20*
Child disclosure	-07***	-.22	-.03	-.21	-1.70
R^2^		.142		.147	
Model 2					
Family structure	.41***	.16	-.03	-.02	3.17***
Parental monitoring	-.01	-.03	-.01	-.06	.00
Conflict with parents	.14***	.18	.02	.04	2.67**
Attachment to parents	.01	.02	-.04***	-.20	2.14*
Child disclosure	-.06***	-.19	-.03	-.10	-1.36
Time spent with friends	.17***	.27	.14***	.31	.82
R^2^		.212		.233	

*p < .05. ** *p* < .01. ***p < .001

^a^Based on the Bonferroni method, threshold of significance, *p* < .008

In the second and final model, where time spent with friends is also included, the model show that for boys, family structure, conflict with parents, child disclosure and time spent with friends are significantly associated with adolescent offending. For girls, it is attachment to parents and time spent with friends. The effect of family structure is significantly greater for boys than for girls (z = 3.17, *p* < .001), as is the effect of conflict with parents (z = 2.67, *p* < .01). The effect of attachment to parents is significantly greater for girls than for boys (z = 2.14, *p* < .05). Following calculations to establish possible differences in the size of the coefficients, the effect of child disclosure was not significantly greater for boys than for girls (z = −1.36). Parental monitoring was not correlated with offending for either boys or girls. The amount of variance explained by this model is 23.3% for girls, as compared with 21.2% for boys.

### Do Family Variables Explain Gender Differences in Offending?

In a second multiple regression analysis (see Table 4), the aim was to examine whether the family variables are able to explain gender differences in adolescent offending. Gender is included as a predictor in the first model, where the results show that gender is significantly associated with offending, indicating that boys offend significantly more than girls. The amount of variance explained by gender is 5.1%.

**Table 4:** OLS multiple regression analysis predicting self-reported offending

Predictors	Model 1		Model 2		Model 3		Model 4	
	*b*	Beta	*b*	Beta	*b*	Beta	*b*	Beta
Gender (1 = girl)	-.45***	-.23	-.46***	-.23	-.47***	-.23	-.44***	-.22
Family structure			.32***	.14	.23**	.10	.17	.08
Parental monitoring					-.02	-.08	-.01	-.04
Conflict with parents					.08***	.13	.07***	.11
Attachment to parents					-.02	-.07	-.02	-.06
Child disclosure					-.05***	-.18	-.05***	-.16
Time spent with friends							-.15***	-.27
R^2^	.051		.071		.173		.238	

Based on the Bonferroni method, threshold of significance, *p* < .007

In the other models, which include the family variables, the gender effect remains relatively unaffected and unchanged throughout. These results indicate that gender differences in adolescent offending are not dependent on, and therefore cannot be explained by, the family variables included in the study. The amount of variance explained in the fourth and final model is 23.8%.

## Discussion and Conclusion

In the present study, the main results show that there are significant differences between boys and girls in the associations between the dimensions of attachment and family interaction and offending. The findings also show that the study variables do not explain the gender gap in adolescent offending, indicating that gender continues to predict offending regardless of these variables.

Altogether, the findings in this study provide some support for social bonding theory (Hirschi 1969), showing that the impact of dimensions of parent-child attachment and interaction are important predictors of young people’s offending. The findings also show that the total variance explained by these family influences is similar among boys and girls. However, the relative importance of the different family influences is not, showing that socialization processes do not seem to be universal across gender. This indicates that, although these family dimensions are of general importance, there are likely to be important gender differences in the way that children bond with their parents, which in turn produce differential involvement in offending. Furthermore, the results of this study point to the importance of including multidimensional measures of relevance to the relationship between parent and child. This enables us to capture more specific distinctions and mechanisms that are important to an understanding of the gendered differences between family influences and adolescent offending.

The seemingly most important differences that emerged in the study indicate that there may be qualitative differences in experiences of and reactions to different aspects of family factors, and/or that boys and girls are susceptible to the different dimensions of relevance to parent-child attachment and interaction to a varying extent. We will clarify this reasoning with a few examples drawn from the results of the present study.

Girls report that they have more conflicts with their parents than boys, although having conflicts with parents was only found to be associated with offending for boys. Further, the girls reported being more closely monitored than the boys. These results need to be understood with reference to the period of adolescence, which, is characterized by aspirations for freedom and independence for example (Steinberg and Silk 2002; Grusec 2011). Bearing this in mind, previous research has shown that parents are generally more worried about their daughters than about their sons, and this has been argued to be an essential reason why girls are also more controlled by parents (Junger-Tas et al. 2004; Svensson 2004). It has also been argued that girls, as a result of this, tend to feel constrained by family ties (ibid.). This might be an explanation for, and may possibly account for part of, the higher levels of conflict with parents reported by girls in the present study. At the same time, a certain level of parent-child conflict in families with adolescents is seen as a necessary part of the process of establishing independence, and the occurrence of parent-child conflicts might not necessarily be defined as producing a negative socialization environment (Formoso et al. 2000).

Potential positive outcomes from certain levels of parent-child conflict, such as learning to manage and deal with conflicts, have been contrasted with the potential negative outcomes, such as the possibility that conflicts might reduce the level of secure parent-child bonding, for example, which would in turn be related to negative behavioral outcomes (Choi et al. 2008). The results of the present study might indicate that boys are more sensitive to the negative consequences of conflict. It has, however, been suggested that when parent-child conflict is considered simultaneously with other family processes, such as bonding with parents, the effect may no longer be significant (Hill et al. 2005). This might contribute a partial understanding of the results presented in this study, in which attachments to parents seem to produce a greater effect among girls than among boys, which is also in line with previous research findings (e.g., Heimer and De Coster 1999). It has been argued that the relationship to parents may have a stronger mediating influence on girls’ offending because girls tend to place a higher value on their relationships during adolescence than boys (Junger-Tas et al. 2004). It has also been noted that girls, at the same time, tend to see their relationships with their parents in a less positive light than boys (ibid.). This might affect measures of social bonding and may have implications for the results produced, since it is possible that the measures and items employed in this study have different effects on the responses of boys and girls respectively (see Hagquist 2007).

Taken together, the results in this study indicate that the mechanisms of social bonding seem to operate in somewhat different ways in relation to boys and girls respectively. For example, the data suggest that for girls the bonding process may operate more effectively through attachment to parents, i.e. through emotional controls.

There are a number of limitations that should be noted in connection with the present study. First, although a number of family variables were included in the analyses, there remain a range of other family-related variables, for example permissive parenting-styles, parental neglect of the child and harsh or inconsistent discipline, that need to be examined more explicitly in order to provide additional nuance to our understanding of this issue. Second, only family variables were included in the study, which means that the study lacks a range of control variables that may be important in explaining the gender gap in adolescent offending, for example self-control. Third, the analyses were only conducted in relation to *parental* bonding, without distinguishing between the relationship with the mother and father respectively. This means that we cannot say anything about differences in levels or about effects on adolescent offending with regard to maternal versus paternal bonding. Fourth, since the data employed in the study are cross-sectional, the study focuses on correlations between family variables and offending among boys and girls. It is not possible to address the issue of temporal causal order. Nor is it possible to say anything about changes in these relationships over time. Fifth, since the results are based on self-report data, this has potential implications for the results that it is important to be aware of. For example, when it comes to adolescents’ perceptions of parenting behavior and parent-child relations, it has been suggested that adolescents tend to report lower levels of e.g. monitoring than their parents (Abar et al. 2015). However, a strong parent-child bond might still be more important when it comes to behavioral outcomes, since this may divert from young persons’ perceptions of parenting behavior.

As regards future research, there is a need to further explore these gender differences on different dimensions that are of relevance for parent-child attachment and also what produces these gendered differences. Socialization patterns within the family need to be analyzed together with individual aspects and structural contexts, such as maturity and gender expectations, for example, in order to produce a more comprehensive picture of this issue. To be able to explain the gender gap in adolescent offending to a greater extent, additional explanatory factors need to be considered, such as school, peers, moral values and self-control, for example. In addition, research within this field could benefit from studying adolescent online activity, since interactions afforded by social media are central to the current generation of adolescents. This could, for example, contribute to a better understanding of *how* time at home is spent. Previous research indicates that girls spend more time on social networking sites and use them more actively than boys (Nesi and Prinstein 2015). This could indicate that even though girls seem to spend more time at home (which is generally seen as a protective factor), they might spend a lot of this time on social media with friends. Findings on whether online activity is associated with negative and/or positive behavioral outcomes and/or well-being are mixed (ibid.). Further research using longitudinal data would also be able to fill several gaps that exist in the literature. For instance, relatively little is known about whether and how the link between family variables and adolescent offending changes over time for boys and girls respectively. There are good reasons to argue that differences in the influence of family on the offending of boys and girls may be partially understood and explained by developmental differences between boys and girls; this is rarely discussed in the literature however.

In conclusion, this study contributes to the existing literature in several ways. For example, the results are not only indicative of real and important differences in the dynamics of patterns of family attachment among boys and girls, they also point to the importance of conducting more nuanced and detailed studies of the different elements of family attachments in order to be able to capture the gendered differences that exist. The findings are important not only in terms of their theoretical relevance, but also in terms of their policy implications, since this knowledge might be important for the development and improvement of family-focused intervention programs that target offending in youth.
